# Digital Health Education for Chronic Lung Disease: Scoping Review

**DOI:** 10.2196/53142

**Published:** 2025-03-18

**Authors:** Chao Sun, Huohuo Dai, Rianne M.J.J. van der Kleij, Rong Li, Hengchang Wu, Cynthia Hallensleben, Sofie H Willems, Niels H Chavannes

**Affiliations:** 1 Department of Public Health and Primary Care Leiden University Medical Center Leiden The Netherlands; 2 National eHealth Living Lab Leiden The Netherlands; 3 School of Pharmacy Hunan University of Chinese Medicine Changsha China; 4 School of Public Health Wuhan University Wuhan China

**Keywords:** digital health education, digital health interventions, chronic lung disease, eHealth, scoping review

## Abstract

**Background:**

Chronic lung disease (CLD) is one of the most prevalent noncommunicable diseases globally, significantly burdening patients and increasing health care expenditures. Digital health education (DHE) is increasingly important in chronic disease prevention and management. However, DHE characteristics and impacts in CLD are rarely reported.

**Objective:**

This study aimed to provide an overview of the existing literature on DHE for CLD, with a focus on exploring the DHE mediums, content, mechanisms, and reported outcomes in patients with CLD.

**Methods:**

We searched PubMed, Web of Science, Embase, PsycINFO, and The Cochrane Library with the assistance of a librarian specialist. Articles were screened by the reviewer team with ASReview (Utrecht University) and EndNote X9 (Clarivate Analytics) based on predefined inclusion and exclusion criteria and the PRISMA-ScR (Preferred Reporting Items for Systematic Reviews and Meta-Analyses extension for Scoping Reviews) checklist. Quality assessment was conducted with the Critical Appraisal Skills Program tool. A descriptive analysis was used to summarize the study characteristics, DHE characteristics, and outcomes.

**Results:**

A total of 22 studies were included in this review with medium or high quality. They were published between 2000 and 2022, showing an increasing publication trend with the year, mostly in developed countries (16/22, 73%). Websites and mobile apps (10/22, 45%) were the most widely used DHE medium. Education on self-management skills of CLD was the primary topic (21/22, 95%), 4/22 (18%) of which mentioned DHE mechanisms. The majority of studies reported positive changes in CLD awareness (14/16, 88%), clinical outcomes (3/6, 50%), DHE feasibility, acceptability, and satisfaction (6/8, 75%), lifestyle outcomes (3/3, 100%), and psychosocial outcomes (7/8, 88%). Only 2 studies reported cost-effectiveness (2/22, 9%).

**Conclusions:**

Despite the heterogeneity of the study situation, some aspects can be concluded. DHE can improve disease awareness and clinical outcomes in patients with chronic lung disease, with good feasibility, acceptability, and satisfaction through different mediums and learning content. There is still relatively little research among people in low- and middle-income countries. Future research should consider the impact on cost-effectiveness, duration, frequency, and theoretical mechanisms of the DHE to maximize the potential impact. It should also be conducted in the context of health services research to better reflect the real world.

## Introduction

Chronic lung disease (CLD) is one of the most prevalent noncommunicable diseases globally, placing a large burden on patients and increasing health care expenditures, primarily including chronic obstructive pulmonary disease (COPD) and asthma. The total number of deaths due to CLD increased by 18% during the past 3 decades, with the highest mortality and disability-adjusted life years in regions with a low sociodemographic index, mainly in Africa, Middle East, Central Asia, Southeast Asia, and South America, also namely low- and middle-income countries (LMICs) [1-3]. Smoking, air pollution, and occupational exposure were the most significant risk factors for CLD [4]. Considering the acute exacerbations [5], complicated comorbidities, and increasing costs of CLD [6], raising disease awareness and promoting healthy lifestyles through health education and management is crucial to improving people’s health conditions among patients with CLD and the general population.

Digital health education (DHE) offers knowledge or skills about health and health care in a timely and cost-effective manner by using all kinds of information and communication technologies (computer-assisted learning, mobile learning, and digital simulation-based learning), which is increasingly important in disease prevention and management [7]. With the accessibility of the internet and the popularity of advanced electronic or digital technology, diverse DHE mediums have emerged, such as websites, multimedia, and mobile apps, providing learners with health information and instant feedback [8-11]. In addition, many studies reported a potential positive impact of digital health interventions on various diseases and mental health status, including improving people’s knowledge, skills, adherence, and other health-related outcomes [12-15]. However, despite the benefits of digital health intervention, the evidence is scarce regarding the characteristics and impact of DHE, especially in patients with CLD.

Currently, suboptimal disease control in patients with CLD is noticed due to their lower medication adherence and incorrect use of inhaler devices, which further contribute to increased exacerbation and hospitalizations [16]. This highlights the importance of DHE to deliver continuous and efficient educational content that helps them better understand their condition and treatment regardless of time and space. However, to the best of our knowledge, no reviews have focused on the characteristics and impact of DHE on CLD. It is essential to conduct a review to understand what aspects of DHE have been explored that could facilitate or hinder their adherence. Furthermore, previous studies on digital health interventions in CLD focused on multidimensional interventions combining disease education with monitoring, coaching, and self-management [17-19]. A review is needed to know if DHE could also lead to significant changes, making a rationale for not giving all patients a multidimensional and, thereby, costly intervention.

Therefore, for this study, we aim to review the existing literature on DHE for CLD, with a focus on exploring the DHE mediums, content, mechanisms, and reported outcomes in patients with CLD. Specifically, we intend to address the following research questions: (1) What are the reported characteristics of the DHE for patients with CLD, in terms of type of DHE mediums, content, duration, frequency, and mechanisms? (2) What outcomes are reported in terms of the impact of DHE on patients’ knowledge of CLD, treatment, management, and health-related outcomes? (3) What is known about the feasibility, acceptability, and patient satisfaction of DHE for patients with CLD?

## Methods

### Study Selection

Search strategies were developed with a medical information specialist from Leiden University Medical Center. Searches were conducted in PubMed, Web of Science, Embase, PsycINFO, and The Cochrane Library combining key search terms “chronic lung disease,” “digital technology,” “health education,” and related terms. To ensure a comprehensive scope by covering all relevant studies on DHE for CLD and capturing a full breadth of the topic, studies were searched from inception to the day we conducted the search (February 26, 2023) without starting time limits. The complete search strategy is available in Multimedia Appendix 1. Literature screening was performed with the PRISMA-ScR (Preferred Reporting Items for Systematic Reviews and Meta-Analyses extension for Scoping Reviews) [20] checklist (Multimedia Appendix 2). Inclusion and exclusion were performed according to the Participants, Intervention, Comparison, and Outcomes (PICO) strategy [21], shown in Textbox 1.

After removing duplicates, one reviewer (CS) used the open-source machine learning tool ASReview Lab19 [22] to screen all the titles and abstracts. Another reviewer (RL) used Endnote X9 to double-screen a random proportion of all articles independently. The choice of 15% was made by the reviewer team to ensure the efficiency and reliability of screening based on ASReview developers’ recommendations [23] and previously published articles that screened the same way [24]. If the agreement rate between the 2 reviewers was less than 80%, an additional random 15% of all titles and abstracts were screened by RL. Title and abstract screening were stopped only when the agreement rate exceeded 80%. Then, the included articles were selected by screening the full text by CS and HD. CS then screened all references of included articles to identify if extra articles could be included. Any discrepancies during the screening process were solved by achieving consensus or discussing with the review team.

**Inclusion and exclusion criteria.**

**Inclusion**

- Participants: people diagnosed with chronic lung disease by a clinical professional.
- Intervention: digital health education focused on chronic lung disease (providing knowledge or skills about health and healthcare by using information and communication technologies).
- Comparison: no restriction.
- Outcome: all outcomes.
- Study design: randomized or nonrandomized controlled trials, qualitative and quantitative studies, mixed methods studies, observational studies.

**Exclusion**

- Not digital health education intervention or blended intervention (eg, interventions that combine education and skills training) that include less than 50% digital health education.
- Studies with subjects <18 years of age.
- Commentaries, reviews, letters, dissertations, editorials, conference proceedings, books, and protocols.
- Not published in English.
- No full text is available.
- Not peer-reviewed articles.

**Justification**

- To ensure a comprehensive review, different study designs were considered: qualitative studies can be used for exploring patients’ experiences, opinions, and attitudes. Quantitative studies can be used to explore the impact of digital health education on chronic lung disease.
- We excluded participants aged less than 18 because there are fewer digital health education interventions directly targeting children with chronic lung disease, but more at their parents, teachers, or pediatric-care professionals.
- Studies with no full text cannot provide complete information about their methods, results, and conclusions.
- A systematic search (query of experts, professional societies, service providers, etc) of non–peer-reviewed publications could not be carried out for reasons of research economics.

### Data Extraction

Reviewer teams (CS, HD, and HW) extracted data with a predesigned standardized data extraction form (Multimedia Appendix 3). Study characteristics (author, publication year, country, profession, study design, sample size, and population levels) were extracted to understand the specific context of each study and assess the relevance, research development, and quality. DHE characteristics (type of DHE medium, content, intervention mechanisms, duration, and frequency) were extracted to understand what aspect of educational platform, content, and behavioral theories were used and how. Outcomes, main findings, and conclusions of included studies were extracted to analyze the impact of DHE on patients with CLD.

### Data Synthesis

Study characteristics and DHE characteristics were summarized using means and percentages where appropriate. Outcome indicators were summarized in 6 categories. CLD awareness evaluated patients’ ability to perceive or to be conscious of CLD or health states (eg, health literacy). Clinical outcomes included physiological and biochemical indicators measured by clinical testing and laboratory equipment (eg, blood pressure, forced expiratory volume [FEV]). Feasibility, acceptability, and satisfaction [25] focused on the design or the development of DHE, evaluating the state or degree of being easily or conveniently used, being subject to acceptance, and the expectations, needs, and pleasure of patients. Lifestyle outcomes included diets, sleep, medication adherence, and physical activity. Cost-effectiveness evaluated the cost expenditure of patients or health care systems on the whole process of disease prevention, treatment, and management. Psychosocial outcomes were observed effects on patients’ emotions, well-being, quality of life (QoL), behavior or social interactions [26]. The impacts of DHE were categorized as positive, negative, or no significant difference based on the extracted results and conclusions. Descriptive analysis was used to summarize all the coded data.

### Quality Assessment

The Critical Appraisal Skills Program (CASP) checklist was used and adapted for appraising the evidence of different study designs [27]. Following CASP tool recommendations, we classified the evidence quality as high (more than 2/3 of questions are answered by “yes”), moderate (more than 1/3 of questions are answered by “yes”), or low (lower than 1/3 of questions are answered by “yes”).

## Results

### Study Characteristics

**Figure 1:**
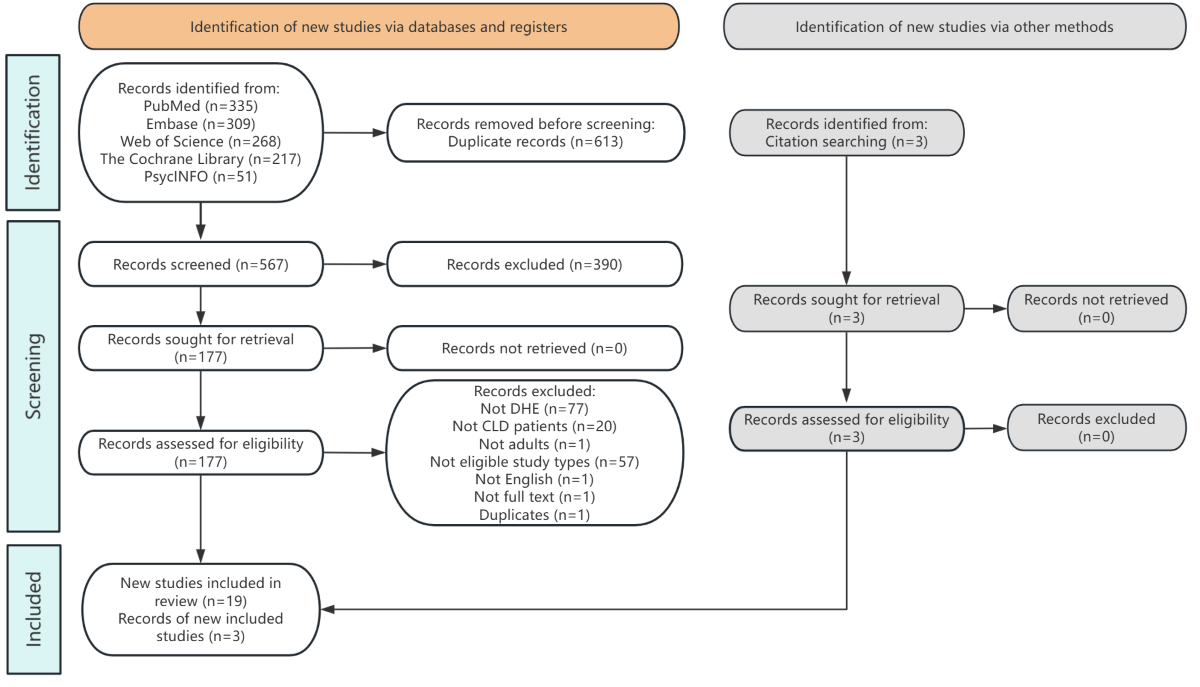
PRISMA-ScR diagram of the review. CLD: chronic lung disease; DHE: digital health education.

**Figure 2:**
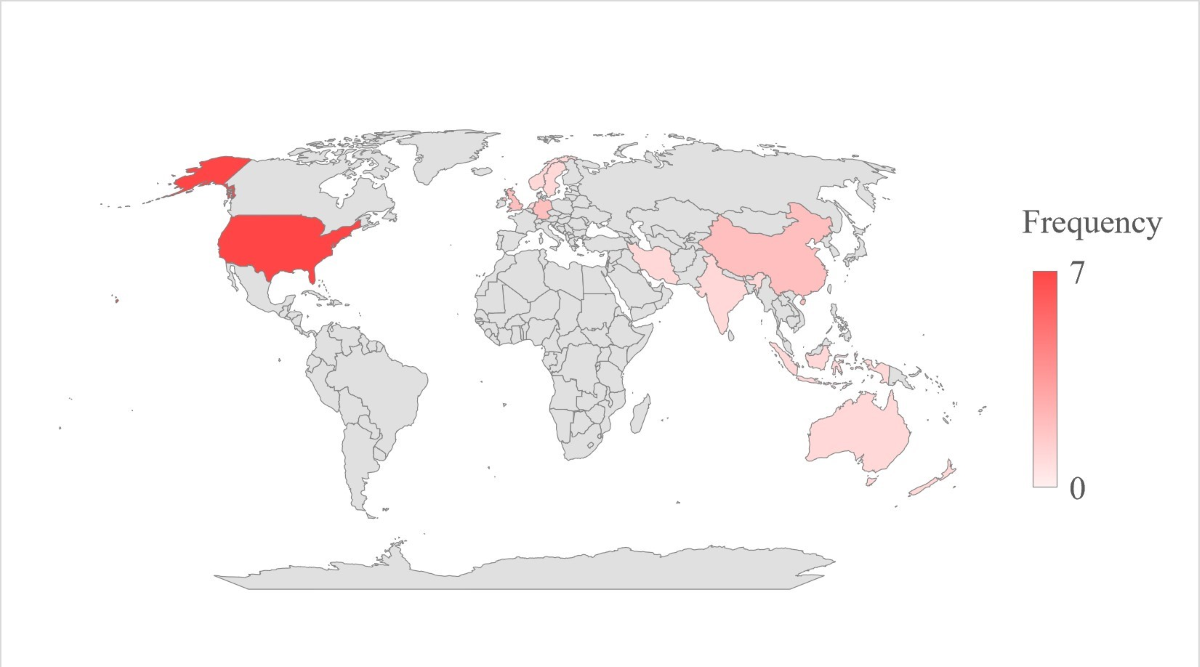
Frequency of publication countries.

A total of 22 articles were included in this scoping review (Figure 1), consisting of 19 interventional studies (9 randomized controlled trials [RCTs], 7 quasi-experimental studies, and 3 prospective interventional studies) and 3 mixed methods studies. These studies were published between 2000 and 2022 in health care or related professions, showing a distinct increasing trend with the highest number after 2020 (n=11). Details of included articles and the trend of publication year were shown in Multimedia Appendix 3. Among these studies, only 27% (6/22) were conducted in LMICs, with China, India, Indonesia, Lebanon, and Iran 1 or 2, respectively (Figure 2). United States accounted for 32% (7/22) of these studies among developed countries. The sample size of the studies varied from 15 to 9452 participants, of which the population level ranged from hospitals, and clinics to general practices, communities, and recruited volunteers. By following the CASP checklist, all studies were medium (n=4) to high quality (n=18). Further details can be found in Table 1 [28-49] and the quality assessment process was described in Multimedia Appendix 4.

**Table 1:** Study characteristics of included articles.

Author	Year	Country	Profession	Study design	Sample size	Population levels	Quality assessment
Nyberg et al [28]	2019	Sweden	Community medicine	Quasi-experiment	83 patients with COPD^a^	Primary care centers	High
Mongiardo et al [29]	2021	United States	Pulmonary care	Prospective interventional study	72 patients with COPD	Outpatient pulmonary clinics	High
Atherton [30]	2000	United States	Analytical services	Quasi-experiment	437 patients with asthma	MyAsthma users	High
Liu et al [31]	2013	China	Nursing	RCT^b^	60 patients with COPD	Volunteer recruitment	Medium
Burns et al [32]	2013	Australia	Health initiatives	Quasi-experiment	51 patients with asthma	Web-based and offline recruitment	High
Wu et al [33]	2017	United States	Public health	Mixed methods study	48 patients with CLD^c^	General medicine service	High
Farzandipour et al [34]	2019	Iran	Health information	Mixed methods study	30 patients with asthma, 10 specialists	Clinic	High
Nohra et al [35]	2022	Lebanon	Public health	Quasi-experiment	15 patients with CLD	Empirical convenience sample	Medium
Newhouse et al [36]	2016	United Kingdom	Primary care	RCT	148 patients with asthma	Primary care practices	Medium
Korsbakke Emtekaer Haesum et al [37]	2016	Norway	Health science and technology	RCT	116 patients with COPD	Telephone recruitment	Medium
Wan et al [38]	2017	United States	Pulmonary care	RCT	109 patients with COPD	General pulmonary clinics	High
Ghozali et al [39]	2022	Indonesia	Pharmacy	RCT	140 patients with asthma	Community	High
Kellerer et al [40]	2022	Germany	General practice	Quasi-experiment	72 patients with asthma	General practices	High
Hsia et al [41]	2020	United States	Medicine	Prospective interventional study	40 patients with asthma	The Montefiore Asthma Center	High
Zhao et al [42]	2020	China	Respiratory medicine	RCT	713 patients with CLD	Hospital	High
Hsia et al [43]	2020	United States	Immunology	Prospective interventional study	60 patients with CLD	Outpatient multispecialty	High
Schnoor et al [44]	2022	The Netherlands	Public health	Quasi-experiment	9452 patients with CLD	Community pharmacies	High
Petrie et al [45]	2012	New Zealand	Psychology	RCT	216 patients with CLD	Flyers and emails recruitment	High
Chan et al [46]	2023	India	Immunology	Mixed methods study	57 patients with CLD	Clinic	High
Moore et al [47]	2009	United Kingdom	Pulmonary rehabilitation	RCT	20 patients with COPD	A program	High
Sobel et al [48]	2009	United States	Internal medicine	Quasi-experiment	130 participants with/without asthma	Organization, education center, clinic	High
Kohler et al [49]	2020	Germany	General practice	RCT	82 patients with asthma	Pulmonary rehabilitation clinic	High

^a^COPD: chronic obstructive pulmonary disease.

^b^RCT: randomized controlled trial.

^c^CLD: chronic lung disease.

### DHE Mediums, Intervention Mechanisms, and Contents

Regarding DHE mediums among the 22 studies, 10 (45%) of them were websites or web-based platforms developed by researchers, along with 6 (27%) studies delivered by mobile applications or tablets, 5 (23%) by video or text messaging, and 1 (5%) by MP3 (Table 2 [28-49]). No more than half (9/22, 41%) of studies conducted DHE for over 3 months. Only 7 studies reported intervention frequency and 4 provided DHE based on the “teach-to-go” strategy, Theory of Planned Behavior, and self-regulation.

**Table 2:** Digital health education characteristics of included articles.

Author	Digital health education medium	Frequency	Duration	Theory
Nyberg et al [28]	An interactive web page	N/A^a^	6 months	N/A
Mongiardo et al [29]	A web-based platform	Daily or weekly	12 months	N/A
Atherton [30]	www.MyAsthma.com	N/A	6 months	N/A
Liu et al [31]	A web-based animated diagram and video	N/A	4 months	N/A
Burns et al [32]	A website	N/A	2 months	Theory of Planned Behavior
Wu et al [33]	Virtual learning platform	N/A	N/A	“teach-to-goal” strategy
Farzandipour et al [34]	A smartphone app	N/A	1 month	N/A
Nohra et al [35]	PowerPoint presentations and videos	Weekly	6 weeks	N/A
Newhouse et al [36]	A multimedia website	N/A	2 weeks	N/A
Korsbakke Emtekaer Haesum et al [37]	A tablet	N/A	4 weeks	N/A
Wan et al [38]	A website	Weekly	3 months	Self-regulation
Ghozali et al [39]	AsmaDroid app	N/A	4 weeks	N/A
Kellerer et al [40]	A web page	N/A	6 months	N/A
Hsia et al [41]	ASTHMAXcel mobile app	One time use	6 months	N/A
Zhao et al [42]	MP3 player	N/A	6 months	N/A
Hsia et al [43]	ASTHMAXcel mobile app	N/A	12 months	N/A
Schnoor et al [44]	A web-based platform SARA^b^	N/A	12 months	N/A
Petrie et al [45]	Text messaging	Irregularly	18 weeks	N/A
Chan et al [46]	Adapted ASTHMAXcel mobile application	N/A	After completing 8 chapters	N/A
Moore et al [47]	Video	4 times a week	6 weeks	N/A
Sobel et al [48]	Multimedia curriculum	Once	Instantly	“teach-to-goal” strategy
Kohler et al [49]	A website	N/A	3 weeks	N/A

^a^N/A: not applicable.

^b^SARA: Service Apothecary Respiratory Advice.

Different contents of DHE in CLD were summarized and divided into several categories (Multimedia Appendix 5). Self-management skills of CLD (21/22, 95%) were the most common educational content, including managing general CLD conditions (20/22, 91%), and exacerbations or asthma attacks (7/22, 32%). Treatment of CLD, disease characteristics, motivational messages, or stories were imparted in 15, 15, and 5 studies, respectively. Specifically, medicine or inhaler education (14/22, 64%) was the most frequently mentioned topic about CLD treatment, followed by breathing exercises or physical activity (5/22, 23%) and psychosocial therapy (3/22, 14%).

### DHE Outcomes for Chronic Lung Disease

After classification and synthesizing, we found that 16/22 (73%) studies explored participants’ CLD awareness. Clinical outcomes (6/22, 27%), DHE feasibility, acceptability, and satisfaction (8/22, 36%), lifestyle (3/22, 14%), cost-effectiveness (2/22, 9%), and psychosocial outcomes (8/22, 36%) were also reported in different studies.

#### CLD Awareness

Fourteen out of 16 studies reported positive changes in CLD awareness. Among them, 9 interventional studies noticed a statistically significant increase (*P*<.05) when evaluating CLD knowledge, literacy, and skills in the intervention group compared with the control group [28,32,35,39,40,42,45,48,49]. In a cohort study, Mongiardo et al [29] found a significant improvement in knowledge about exercise, vaccination, inhaled bronchodilators, and inhaled steroids (*P*<.01) at 12 months among patients with COPD who used a COPD web-based platform that delivers education as part of a physical activity intervention. Similar results were reported in the study by Hsia et al [41,43] by measuring Asthma Knowledge Questionnaire scores among patients with asthma who used ASTHMAXcel—a novel, guidelines-based smartphone app designed to improve patient education and outpatient management of asthma. Chan et al [46] also evaluated an adapted ASTHMAXcel and got the same result using Asthma Knowledge Questionnaire scores among patients with asthma in India. The mean score of patients’ self-management knowledge of patients with asthma was significantly increased to 4.3 (SD 0.56; *P*<.001) after using a smartphone-based app, according to the mixed methods study by Farzandipour et al [34]. When evaluating knowledge and skills after a 6-week home-based educational and telemonitoring program, most of the participants answered correctly [35]. However, Korsbakke Emtekaer Haesum et al [37] reported no statistically significant difference in functional health literacy between the intervention and control group when they focused on the effect of the Telekit—a tele-homecare intervention combining a tablet and software that provided information and instructions on managing COPD. No significant differences in COPD knowledge were noted between the pedometer plus website group and the control group in the RCT by Wan et al [38].

#### Clinical Outcomes

In total, 3 out of 6 studies reported positive clinical outcomes of DHE. These clinical outcomes mainly included changes in the impact of COPD in daily life evaluated by the COPD Assessment Test [28], FEV, forced expiratory volume in 1 second (FEV1)/forced vital capacity (FVC), peak expiratory volume, 6-minute walking distance [31], clinical parameters (systolic and diastolic blood pressure, pulse, FEV1, FVC) [37], dyspnea [38], exacerbation rates, antimycotic treatment difference [44], change in walking capacity [47]. In an RCT, patients with COPD who received a web-based animated diagram and video-guided instruction showed significant improvements in FEV, FEV1/FVC, and peak expiratory volume compared with patients who received conventional education (*P*<.05) [31]. In addition, a significantly lower increase in exacerbation rates over time was found compared to the control participants in participants with asthma but not in individuals with COPD [44]. The walking capacity also showed statistically significant improvements (*P*<.05) within the intervention group measured by the Incremental Shuttle Walk Test after a home exercise video program among patients with moderate to severe COPD [47]. According to Andre et al [28], however, the mean scores of COPD Assessment Test were not highly reduced in patients with mainly moderate COPD, and the difference is statistically insignificant between the baseline and 3-, and 12-month time point. There were also RCTs providing information on managing COPD by a tablet and a website with no statistically significant difference in clinical outcomes, although the intervention duration lasted for several weeks and even 3 months [37,38].

#### DHE Feasibility, Acceptability, and Satisfaction

In total, 6 out of 8 studies indicated a positive effect on DHE feasibility, acceptability, and satisfaction. In the study by Nyberg et al [28], the feasibility analysis of a COPD web page on self-management skills combining texts, pictures, videos, and interactive components was primarily focused on the number of users and the total time they spent on the site during the initial 3 months, showing that 77% (33/43) of patients having access to the COPD Web were considered users and the average total time on the site was 45 minutes, which was quite low. Another feasibility study of a novel experience-based internet intervention to support self-management in chronic asthma was also relatively low, reporting that the median number of logins during 2 weeks was 2, and the median amount of time spent on the site was 17 minutes [36]. In addition, the e-Health Impact Questionnaire scores they used to measure the respondents’ attitudes toward the website were quite similar between groups. Satisfaction, ease-of-use, and usefulness were assessed when analyzing the acceptability of an education website AsthmaWise for older asthma adults, of which 69% (35/51) had decided to visit it to learn more about how to manage their asthma, 94% (48/51) found it useful and almost all participants would recommend it to others [32]. Wu et al [33] developed a virtual teach-to-goal inhaler technique learning module and found that over 80% (39/48) reported that they were likely to use virtual teach-to-goal inhaler technique learning module to gain skills to improve their health. Furthermore, they identified several domains from focus groups for optimal patient use: access, functionality, and quality. In a quasi-experiment, all 15 participants considered the educational and telemonitoring program adequate and acceptable, declaring they had no difficulty explaining it to others and would suggest it to others [35]. The satisfaction rate regarding an audio-educational method (82%, 291/357 vs 66%, 235/356; *P*<.05) and effective feedback (83%, 68/82 vs 53%, 42/80; *P*<.001) were significantly higher in the experimental group than in the control group for patients from respiratory medicine wards [42]. Patients were highly satisfied in the ASTHMAXcel group, scoring on average 27.9 out of 30 points on the satisfaction survey, but no difference was found in either intervention [43]. An average of 50 out of 54 maximum points was noted based on the questionnaire for user interface satisfaction about participants’ postinterventional median satisfaction for the adapted ASTHMAXcel [46].

#### Lifestyle

All (3/3) studies reported positive changes in patients’ lifestyle. When evaluating the effect of a COPD web in promoting self-management of patients, the amount of daily physical activity and time spent in training exercises in the intervention group at 3 months was increased compared with the usual care group, but not statistically different at 12 months [28]. The daily step count in the pedometer plus website group also significantly improved from baseline beginning and maintained to week 13 based on the theory of self-regulation [38]. When comparing medication adherence in the year after the implementation of an eHealth intervention, Service Apothecary Respiratory Advice, both study groups showed an increase in mean medication adherence over time. Especially, it was significantly higher for Service Apothecary Respiratory Advice participants (*P*=.01), according to Schnoor et al [44].

#### Cost-Effectiveness Outcomes

Only 2 studies evaluated the cost-effectiveness of DHE. A study conducted in Lebanon estimated the overall cost of the educational and telemonitoring program was US $200 per patient (educational sessions only) and US $244 per patient (including telemonitoring services), which consisted of the general cost of materials or patients, time nurses spent on patients, hospitalizations and emergency department visits [35]. However, it was difficult for them to evaluate this intervention’s relative cost given the complicated economic situation in Lebanon. The other study reported that all health care utilization endpoints, including asthma-related emergency department visits, hospitalizations, and oral prednisone use, decreased at 4 months and 6 months based on survey results [41].

#### Psychosocial Outcomes

Improved psychosocial outcomes were reported in 7 out of 8 studies. Notably, QoL, usually evaluated by self-reported questionnaire, was the most frequent outcome in these studies. In total, 7 out of 8 studies reported improvement in the intervention group or at the follow-up point [30,31,35,36,38,41,47]. A total of 5 of 15 patients showed lower anxiety and depression scores, and 6 had higher QoL scores compared to baseline measures, according to Rita’s study [35]. Minimal differences of change from baseline between groups were noticed regarding how well patients with asthma manage their condition and self-efficacy of being able to undertake certain activities [36]. Wan et al [38] also compared health-related QoL, exercise self-efficacy, depression, social support, motivation, and confidence to exercise, but no significant differences were noted between groups. All participants showed an increase in health state from baseline based on the physical and mental component of the 36-Item Short Form Survey (including physical and mental dimensions), with a more significant improvement in the intervention group that provided a multimedia website illustrating asthma-related information [36]. No other psychosocial outcomes were analyzed in these studies.

## Discussion

### Principal Findings

Current evidence presents comprehensive characteristics and the potentially positive impact of DHE on CLD. A total of 22 published studies with medium to high quality from 2000 to 2022 were analyzed in this review, including 9 (41%) RCTs, 7 (32%) quasi-experimental studies, 3 (14%) mixed methods studies, and 3 (14%) prospective interventional studies. Most studies (16/22, 73%) were conducted in developed countries. Websites and mobile applications (10/22, 45%) were the most widely used medium to conduct DHE among patients with CLD, followed by mobile apps or tablets, video or text messaging, and MP3. Participants were mainly patients with CLD recruited from hospitals and general practices. No more than half of these studies (9/22, 41%) implemented DHE interventions for more than 3 months, and few (3/22, 14%) were based on theoretical mechanisms when educating the target population. The DHE content varied from a general introduction of CLD characteristics (definition and etiology), and treatment to self-management skills of CLD, motivational messages, and stories for patients and their relatives. Positive impacts were reported among most of these studies, including increased CLD awareness (14/16, 88%), clinical (3/6, 50%), lifestyle (3/3, 100%), psychosocial outcomes (7/8, 88%) and impressive feasibility, satisfaction, and acceptability (6/8, 75%). Only 2 studies reported cost-effectiveness outcomes.

### Comparison With Previous Work

The growing number of publications pertaining to DHE in CLD and the widespread use of websites and mobile apps shed light on an increasingly important role of digital technology in health and health care. As of January 2023, there were 5.16 billion internet users worldwide, over 60% of the global population currently connected to the world wide web with an estimated 192 minutes per day per user spent on the web [50]. In addition, integrating digital technology in health care is revolutionary by providing easy access to health services, improving patient engagement, supporting remote supervision of physical activity regimes at home, delivering timely detection of deteriorating health status, and serving as a cost-saving alternative health service [51]. Although not mentioned in included studies, the integration of artificial technology (AI) in health care, which is another promising DHE medium, holds a significant potential to deliver more efficient and personalized educational content through advanced algorithms and predictive analytics [52,53]. Therefore, users could access a diverse range of health information with better accessibility, efficiency, and interactivity. The increasing trend of exploring the impacts of DHE intervention will continue in future research.

More attention should be paid to implementing and scaling DHE to LMICs and the general population with limited access to digital technology. As reported, the internet penetration rate of China was relatively low at 76% compared with South Korea and Japan, which ranged over the 90% mark, although it is home to the largest digital community in the world, with about 1.07 billion netizens as of 2022 [54]. The same pattern is apparent in Southeast Asian countries with little internet accessibility at home and low self-perceived digital literacy among Southeast Asian youth, thereby reducing health care engagement and hindering health information dissemination for the local people [55]. Larger gains in health can be achieved in these areas due to the huge population underserved, which could also contribute to global health in the future. Furthermore, new opportunities and challenges are presented when scaling up DHE with the increasing aging population and worldwide health problems. Digital technologies play an increasingly important role not only in health education and promotion but also in addressing issues of health equality [56].

Future studies should further elucidate DHE characteristics and mechanisms to ensure long-standing effectiveness and verifiable impacts. First, the predominance of DHE contents focusing on self-management skills of CLD highlights the importance of empowering individuals to take an active role in their own health, which corresponds to the current research trends of chronic diseases [57,58]. Second, successful health education depends on repeatedly using a few messages of proven benefit in many forums [59]. As such, the DHE content and the authenticity of contents should be warranted, of which the latter was rarely mentioned. As an essential health education component, DHE frequency, duration, and intervention theories are closely connected with prospective health outcomes. Several existing models, such as the Unified Theory of Acceptance and Use of Technology, Technology Acceptance Model, Social Cognitive Theory, and Theory of Planned Behavior, have been identified as facilitators of adopting digital health interventions [60]. Specifically, interventions based on the theory of planned behavior tended to have more substantial effects on behavior compared to interventions incorporating fewer techniques [61]. In this review, however, few studies illuminated the frequency (7/22, 32%) and intervention theory (4/22, 18%), indicating a potential gap in understanding how DHE interventions work and the underlying mechanisms that impact patients with CLD and related outcomes.

The effects reported on awareness or knowledge are commonly acknowledged [62-64] because digital technologies can support a wide range of self-directed learning activities, providing learners with diverse resources, information, and network resources that meet their learning needs [9]. Improvement in clinical outcomes suggests the potential of DHE to empower patients to make informed decisions about their health, adhere to treatment regimens, and adopt healthier lifestyles after enhancing their understanding of disease and health conditions. Similar positive effects in QoL and health status were also reported in other reviews, but they mainly focus on digital health interventions [65,66]. Insignificant effects in several studies might be related to the intervention duration or inclusion of participants [37,38,44]. The reported impacts on DHE feasibility, acceptability, and satisfaction shed light on users’ active engagement and willingness with these interventions. These positive findings align with other studies [67,68], indicating that DHE is generally well-received by individuals with CLD and implies promising practicality and scalability in real-world settings.

A limited number of studies reported impacts on lifestyle (n=3) and cost-effectiveness outcomes (n=2) in this review, which needs more evidence to illustrate in the future. The lifestyle outcomes extracted from the included studies were limited to physical activity and medication adherence. According to previous studies, however, a sedentary and unhealthy lifestyle, including smoking, having an unhealthy diet, and insufficient exercise could fuel chronic disease progression by changing interstitial cell behavior [69]. In addition, we noticed that only one study applied behavioral theories but implemented DHE for only 2 months when exploring DHE impacts on lifestyle [32]. However, evidence has shown that a successful digital intervention can influence lifestyle management [70], and the theory of behavior change maintenance can guide the development and evaluation of interventions, thereby promoting sustained change in health behavior and lifestyles [71]. As such, a deeper exploration of specific lifestyles, such as the dietary pattern, types, frequencies, and intensities of exercise and other health-related outcomes should be mapped, with validated and effective theories. Currently, few studies in this review compare the cost or health care utilization after DHE intervention, which guided future cost-effectiveness studies with more accurate and complete data to fill in the gap.

### Recommendation on Future Research

Based on the current review, we recommend that future research and development in DHE for people with CLD should focus on elucidating the characteristics and mechanisms of the DHE intervention, prioritizing less developed areas, investigating more lifestyle or behavioral outcomes, evaluating cost-effectiveness among different DHE mediums and ensuring long-term sustainability, to ultimately improve the effectiveness, scalability of DHE, and the health status of patients with CLD. With the integration of AI in health care, we hope to see more personalized and efficient education content delivered and look forward to studies reporting its potential and effectiveness. Given that several studies were of medium quality based on the CASP tool, we recommend future researchers consider incorporating blinding among participants, investigators, and analysts, as well as addressing confounding factors in both study design and analysis, which will enhance the robustness and reliability.

### Strengths and Limitations

This is the first review to explore the characteristics and impact of DHE on CLD. Diverse studies in this review were synthesized and analyzed under the PRISMA-ScR (Preferred Reporting Items for Systematic Reviews and Meta-Analyses extension for Scoping Reviews) guideline, all showing medium or high-quality evidence with a practical quality assessment tool. The detailed descriptions of DHE and its impact on different outcomes provide an important theoretical basis and new thoughts for further research in DHE. However, this review included studies focusing more on mixed digital health intervention. Despite the potential benefits, whether it is the independent or compounding influence of DHE remains to be discussed further due to the inability of subgroup analysis. The incomplete screening of titles and abstracts by the second reviewer may have introduced potential bias into the review process. The exclusion of non-English articles and studies without full-text access could result in publication bias to some extent. In addition, it is worth mentioning the caution of extrapolating the impact to other DHE mediums, despite recognizing the reliability of health information in these studies. Variations in researchers’ profession might also contribute to heterogeneity in study implementation and reporting.

### Conclusion

Despite the heterogeneity of the study situation, some aspects can be concluded. DHE can improve disease awareness and clinical outcomes among patients with chronic lung disease with good feasibility, acceptability, and satisfaction through different mediums and educational content. There is still relatively limited research among people in low- and middle-income countries. Future research should consider the impacts on cost-effectiveness outcomes, duration, frequency, and theoretical mechanisms of the DHE intervention to maximize the potential effects. It should also be conducted in the context of health services research to better reflect the real-world settings.

## Appendix

Multimedia Appendix 1 Search strategies.

Multimedia Appendix 2 PRISMA-ScR (Preferred Reporting Items for Systematic Reviews and Meta-Analyses extension for Scoping Reviews) checklist.

Multimedia Appendix 3 Data extraction form and included articles.

Multimedia Appendix 4 Quality assessment of included studies.

Multimedia Appendix 5 Overview of digital health education (DHE) content in chronic lung disease (CLD).

## Abbreviations

AI: artificial intelligence
CASP: The Critical Appraisal Skills Program
CLD: chronic lung disease
COPD: chronic obstructive pulmonary disease
DHE: digital health education
FEV: forced expiratory volume
FEV1: forced expiratory volume in one second
FVC: forced vital capacity
LMIC: low- and middle-income country
PICO: Participants, Intervention, Comparison, and Outcomes
PRISMA-ScR: Preferred Reporting Items for Systematic Reviews and Meta-Analyses extension for Scoping Reviews
QoL: quality of life
RCT: randomized controlled trial

## Data Availability

All data generated or analyzed during this study are included in this published article and Multimedia Appendices 1-5.
