# Supplementary material for: Digital Health Education for Chronic Lung Disease: Scoping Review
Source: J Med Internet Res. 2025 Mar 18;27:e53142. doi: 10.2196/53142 (PMC11962326; doi:10.2196/53142)
Supplement: Multimedia Appendix 1 [file jmir_v27i1e53142_app1.docx]

**Search Strategy**

1. Web of Science:

((TI=("Obstructive Lung Diseas*" OR "Obstructive Lungdiseas*" OR "chronic obstructive lung" OR "chronic Lung diseas*" OR "Chronic obstructive pulmonary" OR "Chronic pulmonary" OR "asthma" OR "asthmas" OR "astma" OR "astmas" OR "chronic Bronchitis" OR "Pulmonary Emphysem*" OR "lung Emphysem*" OR "COPD")) AND (TS=("Mobile Application*" OR "Cell Phone*" OR "cellphone*" OR "smartphone*" OR "mobile phone*" OR "Iphone*" OR "Google Play" OR "appstore" OR "DHEA" OR "Patient Portal*" OR "Gamification*" OR "Video Game*" OR "Web Browser*" OR "website*" OR web-site* OR "app" OR "apps" OR "e-training*" OR "telemedicine" OR "tele medicine" OR "telehealth" OR "web based" OR "webbased" OR "internet based" OR "m health" OR "mhealth" OR "mobile health" OR "ehealth" OR "e health" OR "electronic health" OR "virtual-reality" OR "mixed-reality" OR "e-learning*" OR "health website*" OR "digital health*") OR TI=("mobile" OR "online" OR "web" OR "internet" OR "application*" OR "game" OR "games" OR "Internet" OR "software" OR "soft-ware" OR "social-media" OR "computer-assist*" OR "computer-device*" OR "podcast" OR "microcomputer*" OR "micro-computer*" OR "ipad" OR "tablet*" OR "hand-held" OR "handheld")) AND (TS=("awareness" OR "knowledge" OR "health education") OR (TI=(patient* OR health) AND TI=(teach* OR educat* OR learn*)))) NOT (TI=( "child" OR "children" OR "child'*" OR "children'*" OR "schoolchild" OR "schoolchildren" OR "youngster" OR "youngsters" OR "boy" OR "boys" OR "girl" OR "girls" OR "adolescen*" OR "pre-adolescent" OR "pre-adolescents" OR "pre-adolescence" OR "schoolage" OR "schoolboy" OR "schoolboys" OR "schoolgirl" OR "schoolgirls" OR "pre-puber" OR "pre-pubers" OR "pre-puberty" OR "prepuber" OR "prepubers" OR "prepuberty" OR "puber" OR "pubers" OR "puberty" OR "teenager" OR "teenagers" OR "teens" OR "youth" OR "youths" OR "underaged" OR "under-aged" OR "Pediatric" OR "Pediatrics" OR "Paediatric" OR "Paediatrics" OR "Review"[Publication Type] OR "Systematic Review" [Publication Type] OR "systematic review*" OR "scoping review*" OR "umbrella review*" OR "narrative review*" OR "metaanalysis" OR "metaanalyses" OR "meta-analysis" OR "meta-analyses" OR "commentar*" OR "dissertation*" OR "letter" OR "editorial" OR "conference" OR "books" OR "books" OR "abstract" OR "abstracts"))

1. PubMed

(("Lung Diseases, Obstructive"[majr] OR "Obstructive Lung Diseas*"[ti] OR "Obstructive Lungdiseas*"[ti] OR "chronic obstructive lung"[ti] OR "chronic Lung diseas*"[ti] OR "Chronic obstructive pulmonary"[ti] OR "Chronic pulmonary"[ti] OR "asthma"[ti] OR "asthmas"[ti] OR "astma"[ti] OR "astmas"[ti] OR "chronic Bronchitis"[ti] OR "Pulmonary Emphysem*"[ti] OR "lung Emphysem*"[ti] OR "COPD"[ti]) AND ("Mobile Applications"[Mesh] OR "Cell Phone"[Mesh] OR "Digital Technology"[Mesh] OR "Patient Portals"[Mesh] OR "Gamification"[Mesh] OR "Video Games"[Mesh] OR "Web Browser"[Mesh] OR "Mobile Application*"[tiab] OR "Cell Phone*"[tiab] OR "cellphone*"[tiab] OR "smartphone*"[tiab] OR "mobile phone*"[tiab] OR "Iphone*"[tiab] OR "Google Play"[tiab] OR "appstore"[tiab] OR "DHEA"[tiab] OR "Patient Portal*"[tiab] OR "Gamification*"[tiab] OR "Video Game*"[tiab] OR "Web Browser*"[tiab] OR "website*"[tiab] OR web-site*[tiab] OR "app"[tiab] OR "apps"[tiab] OR "e-training*"[tiab] OR "telemedicine"[tiab] OR "tele medicine"[tiab] OR "telehealth"[tiab] OR "web based"[tiab] OR "webbased"[tiab] OR "internet based"[tiab] OR "m health"[tiab] OR "mhealth"[tiab] OR "mobile health"[tiab] OR "ehealth"[tiab] OR "e health"[tiab] OR "electronic health"[tiab] OR "virtual-reality"[tiab] OR "mixed-reality"[tiab] OR "mobile"[ti] OR "online"[ti] OR "web"[ti] OR "internet"[ti] OR "application*"[ti] OR "game"[ti] OR "games"[ti] OR "Internet"[ti] OR "software"[ti] OR "soft-ware"[ti] OR "social-media"[ti] OR "computer-assist*"[ti] OR "computer-device*"[ti] OR "podcast"[ti] OR "microcomputer*"[ti] OR "micro-computer*"[ti] OR "ipad"[ti] OR "tablet*"[ti] OR "hand-held"[ti] OR "handheld"[ti] OR "digital health*"[tiab] OR "e-learning*"[tiab] OR "health website*"[tiab]) AND ("Awareness"[mesh] OR "Health Education"[Mesh] OR "Knowledge"[Mesh] OR "Patient Medication Knowledge"[Mesh] OR "Health Knowledge, Attitudes, Practice"[Mesh] OR "Patient Education as Topic"[Mesh] OR "awareness"[tiab] OR "knowledge"[tiab] OR "health education"[tiab] OR ((patient*[ti] OR health[ti]) AND (teach*[ti] OR educat*[ti] OR learn*[ti])))) NOT ( "child"[ti] OR "children"[ti] OR "child'*"[ti] OR "children'*"[ti] OR "schoolchild"[ti] OR "schoolchildren"[ti] OR "youngster"[ti] OR "youngsters"[ti] OR "boy"[ti] OR "boys"[ti] OR "girl"[ti] OR "girls"[ti] OR "adolescen*"[ti] OR "pre-adolescent"[ti] OR "pre-adolescents"[ti] OR "pre-adolescence"[ti] OR "schoolage"[ti] OR "schoolboy"[ti] OR "schoolboys"[ti] OR "schoolgirl"[ti] OR "schoolgirls"[ti] OR "pre-puber"[ti] OR "pre-pubers"[ti] OR "pre-puberty"[ti] OR "prepuber"[ti] OR "prepubers"[ti] OR "prepuberty"[ti] OR "puber"[ti] OR "pubers"[ti] OR "puberty"[ti] OR "teenager"[ti] OR "teenagers"[ti] OR "teens"[ti] OR "youth"[ti] OR "youths"[ti] OR "underaged"[ti] OR "under-aged"[ti] OR "Pediatric"[ti] OR "Pediatrics"[ti] OR "Paediatric"[ti] OR "Paediatrics"[ti] OR "Review"[Publication Type] OR "Systematic Review" [Publication Type] OR "systematic review*"[ti] OR "scoping review*"[ti] OR "umbrella review*"[ti] OR "narrative review*"[ti] OR "metaanalysis"[ti] OR "metaanalyses"[ti] OR "meta-analysis"[ti] OR "meta-analyses"[ti] OR "Meta-Analysis"[Publication Type] OR "Letter"[Publication Type] OR "Editorial"[Publication Type] OR "Comment"[Publication Type] OR "commentar*"[ti] OR "dissertation*"[ti] OR "letter"[ti] OR "editorial"[ti] OR "conference"[ti] OR "books"[ti] OR "books"[ti] OR "abstract"[ti] OR "abstracts"[ti])

1. Embase

((exp *"obstructive lung disease"/ OR "Obstructive Lung Diseas*".ti. OR "Obstructive Lungdiseas*".ti. OR "chronic obstructive lung".ti. OR "chronic Lung diseas*".ti. OR "Chronic obstructive pulmonary".ti. OR "Chronic pulmonary".ti. OR "asthma".ti. OR "asthmas".ti. OR "astma".ti. OR "astmas".ti. OR "chronic Bronchitis".ti. OR "Pulmonary Emphysem*".ti. OR "lung Emphysem*".ti. OR "COPD".ti.) AND (exp *"mobile application"/ OR exp *"mobile phone"/ OR exp *"digital technology"/ OR exp *"gamification"/ OR exp *"video game"/ OR exp *"web browser"/ OR "Mobile Application*".ti. OR "Cell Phone*".ti. OR "cellphone*".ti. OR "smartphone*".ti. OR "mobile phone*".ti. OR "Iphone*".ti. OR "Google Play".ti. OR "appstore".ti. OR "DHEA".ti. OR "Patient Portal*".ti. OR "Gamification*".ti. OR "Video Game*".ti. OR "Web Browser*".ti. OR "website*".ti. OR web-site*.ti. OR "app".ti. OR "apps".ti. OR "e-training*".ti. OR "telemedicine".ti. OR "tele medicine".ti. OR "telehealth".ti. OR "web based".ti. OR "webbased".ti. OR "internet based".ti. OR "m health".ti. OR "mhealth".ti. OR "mobile health".ti. OR "ehealth".ti. OR "e health".ti. OR "electronic health".ti. OR "virtual-reality".ti. OR "mixed-reality".ti. OR "mobile".ti. OR "online".ti. OR "web".ti. OR "internet".ti. OR "application*".ti. OR "game".ti. OR "games".ti. OR "Internet".ti. OR "software".ti. OR "soft-ware".ti. OR "social-media".ti. OR "computer-assist*".ti. OR "computer-device*".ti. OR "podcast".ti. OR "microcomputer*".ti. OR "micro-computer*".ti. OR "ipad".ti. OR "tablet*".ti. OR "hand-held".ti. OR "handheld".ti. OR "digital health*".ti. OR "e-learning*".ti. OR "health website*".ti.) AND (exp "awareness"/ OR exp "health education"/ OR exp "knowledge"/ OR exp "patient education"/ OR "awareness".ti,ab. OR "knowledge".ti,ab. OR "health education".ti,ab. OR ((patient*.ti. OR health.ti.) AND (teach*.ti. OR educat*.ti. OR learn*.ti.)))) NOT ( "child".ti. OR "children".ti. OR "child'*".ti. OR "children'*".ti. OR "schoolchild".ti. OR "schoolchildren".ti. OR "youngster".ti. OR "youngsters".ti. OR "boy".ti. OR "boys".ti. OR "girl".ti. OR "girls".ti. OR "adolescen*".ti. OR "pre-adolescent".ti. OR "pre-adolescents".ti. OR "pre-adolescence".ti. OR "schoolage".ti. OR "schoolboy".ti. OR "schoolboys".ti. OR "schoolgirl".ti. OR "schoolgirls".ti. OR "pre-puber".ti. OR "pre-pubers".ti. OR "pre-puberty".ti. OR "prepuber".ti. OR "prepubers".ti. OR "prepuberty".ti. OR "puber".ti. OR "pubers".ti. OR "puberty".ti. OR "teenager".ti. OR "teenagers".ti. OR "teens".ti. OR "youth".ti. OR "youths".ti. OR "underaged".ti. OR "under-aged".ti. OR "Pediatric".ti. OR "Pediatrics".ti. OR "Paediatric".ti. OR "Paediatrics".ti. OR exp "systematic review"/ OR exp "peer review"/ OR exp "systematic review (topic)"/ OR "systematic review*".ti. OR "scoping review*".ti. OR "umbrella review*".ti. OR "narrative review*".ti. OR "metaanalysis".ti. OR "metaanalyses".ti. OR "meta-analysis".ti. OR "meta-analyses".ti. OR exp "meta analysis"/OR exp "letter"/ OR exp "editorial"/ OR exp conference paper/ OR "commentar*".ti. OR "dissertation*".ti. OR "letter".ti. OR "editorial".ti. OR "conference".ti. OR "books".ti. OR "books".ti. OR "abstract".ti. OR "abstracts".ti.)

1. PsycINFO

((MM "Chronic Obstructive Pulmonary Disease" OR MM "Bronchial Disorders" OR MM "Pulmonary Emphysema" OR TI "Obstructive Lung Diseas*" OR TI "Obstructive Lungdiseas*" OR TI "chronic obstructive lung" OR TI "chronic Lung diseas*" OR TI "Chronic obstructive pulmonary" OR TI "Chronic pulmonary" OR TI "asthma" OR TI "asthmas" OR TI "astma" OR TI "astmas" OR TI "chronic Bronchitis" OR TI "Pulmonary Emphysem*" OR TI "lung Emphysem*" OR TI "COPD") AND (DE "Mobile Applications" OR DE "Mobile Phones" OR DE "Smartphones" OR DE "Digital Technology" OR DE "Computer Games" OR DE "Websites" OR TX "Mobile Application*" OR TX "Cell Phone*" OR TX "cellphone*" OR TX "smartphone*" OR TX "mobile phone*" OR TX "Iphone*" OR TX "Google Play" OR TX "appstore" OR TI "DHEA" OR AB "DHEA" OR TX "Patient Portal*" OR TX "Gamification*" OR TX "Video Game*" OR TX "Web Browser*" OR TX "website*" OR TX "web-site*" OR TX "app" OR TX "apps" OR TX "e-training*" OR TX "telemedicine" OR TX "tele medicine" OR TX "telehealth" OR TX "web based" OR TX "webbased" OR TX "internet based" OR TX "m health" OR TX "mhealth" OR TX "mobile health" OR TX "ehealth" OR TX "e health" OR TX "electronic health" OR TX "virtual-reality" OR TX "mixed-reality" OR TI "mobile" OR TI "online" OR TI "web" OR TI "internet" OR TI "application*" OR TI "game" OR TI "games" OR TI "Internet" OR TI "software" OR TI "soft-ware" OR TI "social-media" OR TI "computer-assist*" OR TI "computer-device*" OR TI "podcast" OR TI "microcomputer*" OR TI "micro-computer*" OR TI "ipad" OR TI "tablet*" OR TI "hand-held" OR TI "handheld" OR TX "digital health*" OR TX "e-learning*" OR TX "health website*") AND (DE "Awareness" OR DE "Health Awareness" OR DE "Health Education" OR DE "Knowledge (General)" OR DE "Health Knowledge" OR DE "Knowledge Transfer" OR DE "Knowledge Level" OR DE "Knowledge Management" OR DE "Knowledge Transfer" OR DE "Client Education" OR TX "awareness" OR TX "knowledge" OR TX "health education" OR ((TI patient* OR TI health) AND (TI teach* OR TI educat* OR TI learn*)))) NOT (TI "child" OR TI "children" OR TI "child'*" OR TI "children'*" OR TI "schoolchild" OR TI "schoolchildren" OR TI "youngster" OR TI "youngsters" OR TI "boy" OR TI "boys" OR TI "girl" OR TI "girls" OR TI "adolescen*" OR TI "pre-adolescent" OR TI "pre-adolescents" OR TI "pre-adolescence" OR TI "schoolage" OR TI "schoolboy" OR TI "schoolboys" OR TI "schoolgirl" OR TI "schoolgirls" OR TI "pre-puber" OR TI "pre-pubers" OR TI "pre-puberty" OR TI "prepuber" OR TI "prepubers" OR TI "prepuberty" OR TI "puber" OR TI "pubers" OR TI "puberty" OR TI "teenager" OR TI "teenagers" OR TI "teens" OR TI "youth" OR TI "youths" OR TI "underaged" OR TI "under-aged" OR TI "Pediatric" OR TI "Pediatrics" OR TI "Paediatric" OR TI "Paediatrics" OR TI "systematic review*" OR TI "scoping review*" OR TI "umbrella review*" OR TI "narrative review*" OR TI "metaanalysis" OR TI "metaanalyses" OR TI "meta-analysis" OR TI "meta-analyses" OR TI "commentar*" OR TI "dissertation*" OR TI "letter" OR TI "editorial" OR TI "conference" OR TI "books" OR TI "books" OR TI "abstract" OR TI "abstracts")

1. The Cochrane Library

((("Obstructive Lung Diseas*" OR "Obstructive Lungdiseas*" OR "chronic obstructive lung" OR "chronic Lung diseas*" OR "Chronic obstructive pulmonary" OR "Chronic pulmonary" OR "asthma" OR "asthmas" OR "astma" OR "astmas" OR "chronic Bronchitis" OR "Pulmonary Emphysem*" OR "lung Emphysem*" OR "COPD"):ti,ab,kw) AND (("Mobile Application*" OR "Cell Phone*" OR "cellphone*" OR "smartphone*" OR "mobile phone*" OR "Iphone*" OR "Google Play" OR "appstore" OR "DHEA" OR "Patient Portal*" OR "Gamification*" OR "Video Game*" OR "Web Browser*" OR "website*" OR web-site* OR "app" OR "apps" OR "e-training*" OR "telemedicine" OR "tele medicine" OR "telehealth" OR "web based" OR "webbased" OR "internet based" OR "m health" OR "mhealth" OR "mobile health" OR "ehealth" OR "e health" OR "electronic health" OR "virtual-reality" OR "mixed-reality" OR "e-learning*" OR "health website*" OR "digital health*"):ti,ab,kw OR ("mobile" OR "online" OR "web" OR "internet" OR "application*" OR "game" OR "games" OR "Internet" OR "software" OR "soft-ware" OR "social-media" OR "computer-assist*" OR "computer-device*" OR "podcast" OR "microcomputer*" OR "micro-computer*" OR "ipad" OR "tablet*" OR "hand-held" OR "handheld"):ti) AND (("awareness" OR "knowledge" OR "health education"):ti,ab,kw OR ((patient* OR health):ti AND (teach* OR educat* OR learn*):ti))) NOT ("child" OR "children" OR "child'*" OR "children'*" OR "schoolchild" OR "schoolchildren" OR "youngster" OR "youngsters" OR "boy" OR "boys" OR "girl" OR "girls" OR "adolescen*" OR "pre-adolescent" OR "pre-adolescents" OR "pre-adolescence" OR "schoolage" OR "schoolboy" OR "schoolboys" OR "schoolgirl" OR "schoolgirls" OR "pre-puber" OR "pre-pubers" OR "pre-puberty" OR "prepuber" OR "prepubers" OR "prepuberty" OR "puber" OR "pubers" OR "puberty" OR "teenager" OR "teenagers" OR "teens" OR "youth" OR "youths" OR "underaged" OR "under-aged" OR "Pediatric" OR "Pediatrics" OR "Paediatric" OR "Paediatrics" OR "Review" OR "Systematic Review" OR "systematic review*" OR "scoping review*" OR "umbrella review*" OR "narrative review*" OR "metaanalysis" OR "metaanalyses" OR "meta-analysis" OR "meta-analyses" OR "commentar*" OR "dissertation*" OR "letter" OR "editorial" OR "conference" OR "books" OR "books" OR "abstract" OR "abstracts"):ti
