# Supplementary figures and images for: Digital Health Education for Chronic Lung Disease: Scoping Review
Source: J Med Internet Res. 2025 Mar 18;27:e53142. doi: 10.2196/53142 (PMC11962326; doi:10.2196/53142)

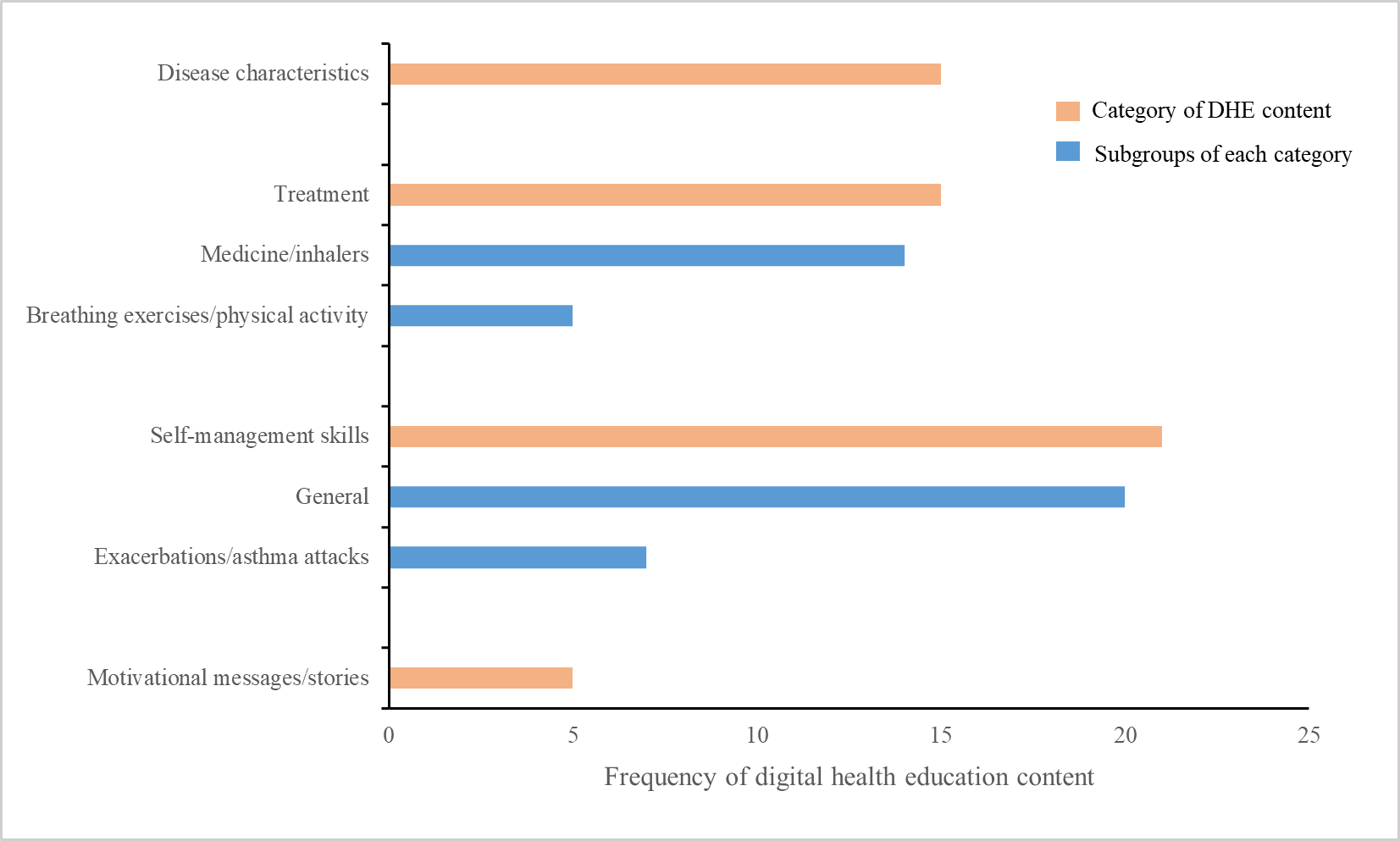

Supplement: Multimedia Appendix 5 [file jmir_v27i1e53142_app5.png]
